# ENSO feedback drives variations in dieback at a marginal mangrove site

**DOI:** 10.1038/s41598-021-87341-5

**Published:** 2021-04-14

**Authors:** S. M. Hickey, B. Radford, J. N. Callow, S. R. Phinn, C. M. Duarte, C. E. Lovelock

**Affiliations:** 1 The School of Agriculture and Environment, Faculty of Science, University of Western Australia, Perth, WA Australia; 2 The Oceans Institute, University of Western Australia, Perth, WA Australia; 3 Australian Institute of Marine Science (AIMS), Crawley, WA Australia; 4 Remote Sensing Research Centre, School of Earth and Environmental Sciences, The University of Queensland, Brisbane, QLD Australia; 5 Red Sea Research Center, King Abdullah University of Science and Technology, Thuwal, Saudi Arabia; 6 School of Biological Sciences, The University of Queensland, Brisbane, QLD Australia

**Keywords:** Plant ecology, Ecology, Ecology, Environmental sciences, Environmental impact, Climate change

## Abstract

Ocean–atmosphere climatic interactions, such as those resulting from El Niño Southern Oscillation (ENSO) are known to influence sea level, sea surface temperature, air temperature, and rainfall in the western Pacific region, through to the north-west Australian Ningaloo coast. Mangroves are ecologically important refuges for biodiversity and a rich store of blue carbon. Locations such as the study site (Mangrove Bay, a World Heritage Site within Ningaloo Marine Park and Cape Range National Park) are at the aridity range-limit which means trees are small in stature, forests small in area, and are potentially susceptible to climate variability such as ENSO that brings lower sea level and higher temperature. Here we explore the relationship between mangrove dieback, and canopy condition with climatic variables and the Southern Oscillation Index (SOI)—a measure of ENSO intensity, through remote sensing classification of Landsat satellite missions across a 29 year period at a north-west Australian site. We find that the SOI, and seasonal mean minimum temperature are strongly correlated to mangrove green canopy (as indicator of live canopy) area. This understanding of climate variations and mangrove temporal heterogeneity (patterns of abundance and condition) highlights the sensitivity and dynamics of this mangrove forest and recommends further research in other arid and semi-arid tropical regions at mangrove range-limits to ascertain the extent of this relationship.

## Introduction

The global climate system controls ocean and atmosphere interactions, influencing sea surface temperature (SST), air temperature, rainfall, and wind^1^. Such climatic variables have been reported as drivers of mangrove variability at local and regional scales^2^. Most notably, temperature, salinity and precipitation have been consistently reported as constraining abiotic variables in mangrove forest condition, and distribution^3^, and when altered have resulted in tree mortality^4^, change in species composition, and loss or reduction in function (e.g., ability to store carbon)^5^.

Mangroves in arid ecoregions are often characterised by trees that are small in stature and limited in areal cover, yet they are ecologically important refuges for biodiversity in the region^3^, important blue carbon sinks^5^, and act as a buffer for coastal protection^6^. *Avicennia* spp*.* covers the largest range, exhibiting a broad tolerance to such abiotic conditions. Whilst salt-tolerant, mangroves are susceptible to high salinity and as such are vulnerable to the effects of climatic changes, including the frequency and intensity of drought events^7,8^. However, extreme conditions can result in physiological thresholds being reached or exceeded, thereby impacting metabolic processes^9^ and causing physiological stress or mortality, as has been observed during extreme drought events, or post hydrological enforcements (e.g., tide barriers)^10^.

Coupled ocean–atmosphere phenomena, such as the El Niño Southern Oscillation (ENSO) influence sea level, SST, air temperature, and rainfall in the western Pacific region, with effects noted at various regions across the globe^11^. In north-west Australia, the Ningaloo coast experiences known fluctuations in sea level, and SST. The weakening of the equatorial trade winds during El Niño events results in the shoaling of the thermocline in the western Pacific, and in turn lower sea levels caused by cooler waters on the west Australian coastline^12,13^. The reverse occurs during La Niña events. Therefore, during an El Niño event the mangroves of north-western Australia are subjected to lower sea levels^14^. The minimal freshwater input in these semi-arid environments means they are potentially subjected to higher saline conditions in the upper tidal and edge locations, which can lead to loss of mangrove canopy and mortality. For example, Drexler and Ewel^15^ reported that during the 1997/98 El Niño, salinity in a Micronesian mangrove forest increased, while rainfall decreased and the water table lowered, limiting freshwater in the forest and resulting in a decline in mangrove growth rate^15^. Furthermore, the largest mangrove mortality observed occurred in remote northern Australia and coincided with the 2015/16 El Niño^4,7,16^. This large-scale dieback event has been linked to lower water levels and high salinity^4,7,16^, resulting in changes to the geochemical composition of the trees and sediment pre and post the mortality^7^.

Mangroves provide critical habitat, coastal protection, and store vast amounts of atmospheric carbon. The frequency, and duration of El Niño events is expected to intensify with increased global temperatures^17^. Variations in climatic events, and in turn the variables they influence, such as sea level, and temperature, have repercussions for the marine environment. ENSO events have shown to influence juvenile fish recruitment, and whale shark aggregation on the Ningaloo coast (north-west Australian coastline)^18^. At Mangrove Bay, within Ningaloo Marine Park and Cape Range National Park, two known mangrove dieback events coincided with El Niño events, and associated lower sea levels, that resulted in higher soil salinity^5,19^. Understanding how variation in climate and environment influences the temporal heterogeneity (patterns of abundance and condition) of mangrove forests remains an ongoing priority, especially in a semi-arid climatic zone that is at the upper mangrove abiotic thresholds (e.g., temperature and salinity).

Remotely sensed spatiotemporal datasets enable large amounts of data to be interrogated at local (site) to global scales^20^. In particular, Landsat imagery provides a regular, consistent and recent history of mangrove change that can be used to compare with environmental data over time. The distinct spectral signature of mangroves means remotely sensed data can enable their extent and some of their structural and physiological properties to be mapped^17–22^. Previous studies have tended to focus on specific locations within the mangrove canopy and not across the entire mangrove area, or on a singular event, and not across a significant time-period covering both impact and recovery of mangrove canopy alongside multiple ENSO phases, we aim to do this here. The objective of this study is to investigate the relationship between mangrove canopy extent, with climatic variables (air temperature, precipitation) and the Southern Oscillation Index (SOI). We also explore the Normalised Difference Vegetation Index (NDVI) as a proxy to determine the spatial variation in the canopy condition through the study period. The SOI provides an indication of the intensity of ENSO conditions^23^ by measuring the strength of the Walker Circulation^23^, which refers to the wind circulation in same tropical region (Julian and Chervin 1978).

**Figure 1:**
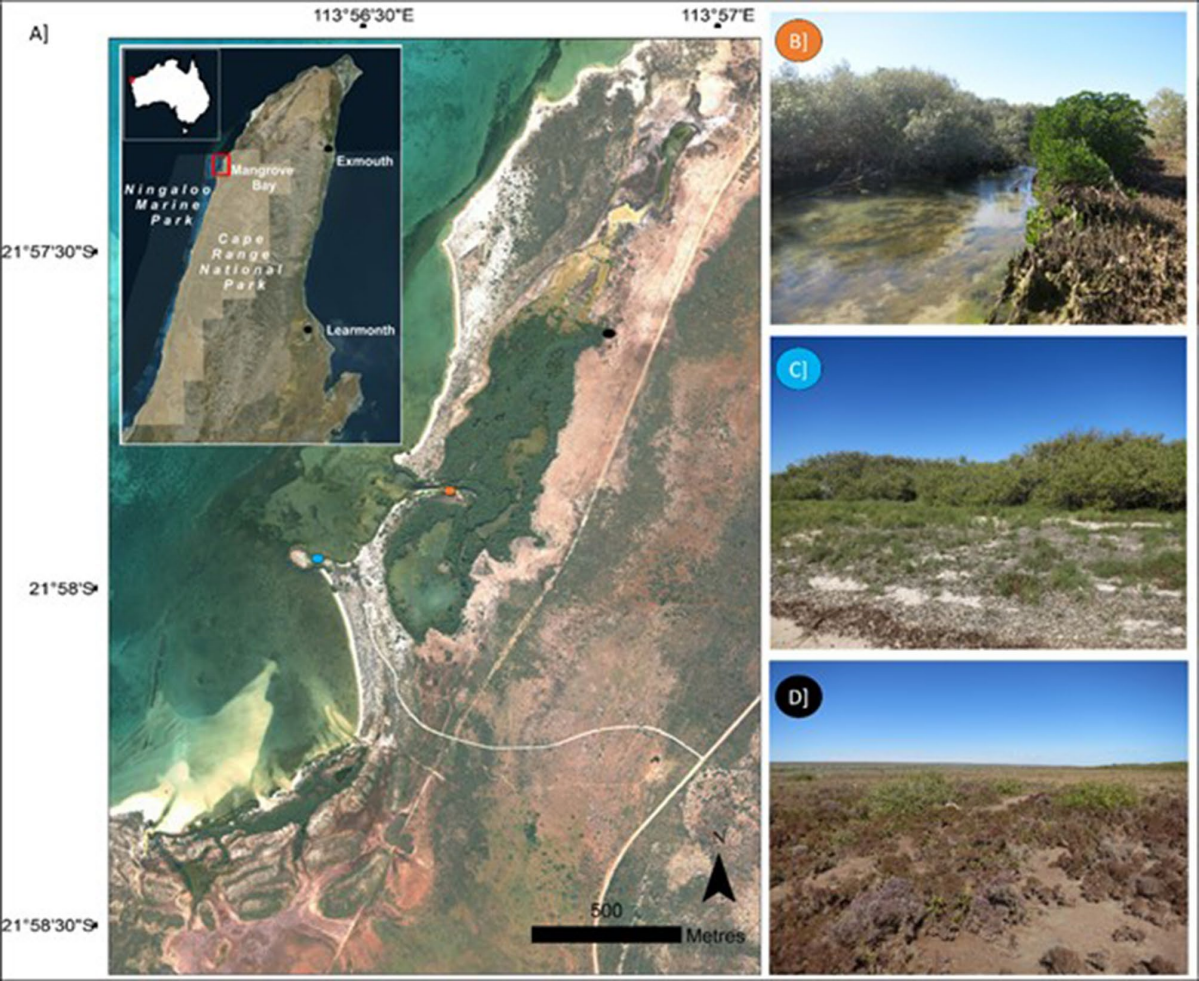
(**A**) Mangrove Bay, a small lagoon fringing coral reefs lies within Ningaloo Marine Park and Cape Range National Park, in north-western Australia (inset map). The site is predominately *Avicennia marina*, with some *Rhizophora stylosa* occurring around the lagoon (**B**), it is bordered by small shrubs and grasses within an arid bioregion (**C**, **D**). Figure created in ArcGIS v10.6. Photo (**C**, **D**) credit: S. Hickey. Aerial imagery provided by WA Department of Biodiversity, Conservation and Attractions.

To examine the relationship between mangroves in north-west Australia, climatic variables and the SOI we first determine mangrove canopy area for each time-point across the observed period (1987 to 2016) through classifying green mangrove canopy from the spectral properties in the Landsat image series for Mangrove Bay (Fig. 1) using a supervised classification method. Mangrove Bay was specifically chosen as it represents a marginal environment where environmental gradients limiting mangrove growth are expressed in a small accessible geographic area. We use Generalised Additive Mixed Models (GAMMs) to ascertain the relationship between climatic variables and the SOI, with mangrove canopy area. We then explore the spatial heterogeneity of the canopy using the NDVI as a proxy to determine the spatial variation in canopy condition as observed across the time-period, as a means to illustrate where change in condition has occurred within the canopy. NDVI is related to condition in mangroves, with higher values indicating healthy, dense forests^24^.

Mangrove Bay is located in a semi-arid environment in the Pilbara region of NW Australia within Ningaloo Marine Park and Cape Range National Park (Fig. 1). It is dominated by *Avicennia marina*, though a range of other species make minor contributions to the canopy. The region can experience rapid heavy rainfall due to tropical cyclones occurring December–April, though a higher incidence of rainy days generally occurs in the winter months. Mean annual temperature ranges from 18 to 32 °C, with higher temperatures averaging 37 °C December-February. Despite its small size, Mangrove Bay is of high ecological importance due to its role in supporting biodiversity in the region as one of the southernmost mangrove stands in Western Australia (Cassata and Collins 2008; Reef et al. 2014), and an important ecotone near the tropical and temperate marine transition zone.

## Results

### Mangrove classification

**Figure 2:**
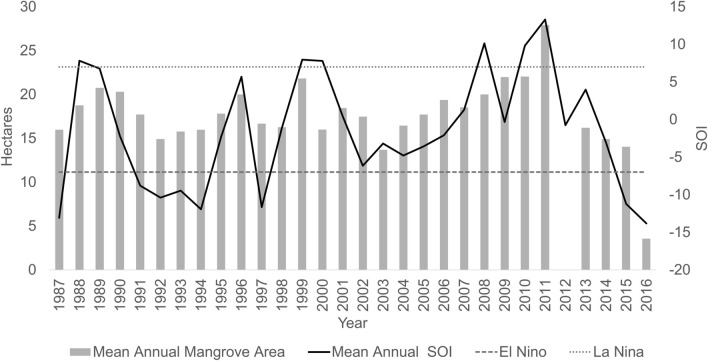
Annual mean mangrove area determined from the green canopy spectral classification (left axis). The Southern Oscillation Index (SOI) annual average for the study period (right axis). A value of -7 or less indicates El Nino conditions, whilst + 7 is indicative of La Nina conditions. The oscillating green mangrove canopy and SOI have an overlapping trend.

The mangrove canopy area derived from supervised classification of the Landsat image series (5TM, 7 ETM, 8OLI) from 213 time-points (months) varied temporally (Fig. 2). The classification was based on green canopy and represents the live (as represented by green leaves) mangrove canopy area from spectral properties. Mean annual canopy area identified illustrated an oscillating pattern (Fig. 2) across the years, which correlated with the SOI (Fig. 2). Temperature and precipitation varied across the years and were also correlated with canopy area (SI1).

### Mangroves, climatic variables and the SOI

**Figure 3:**
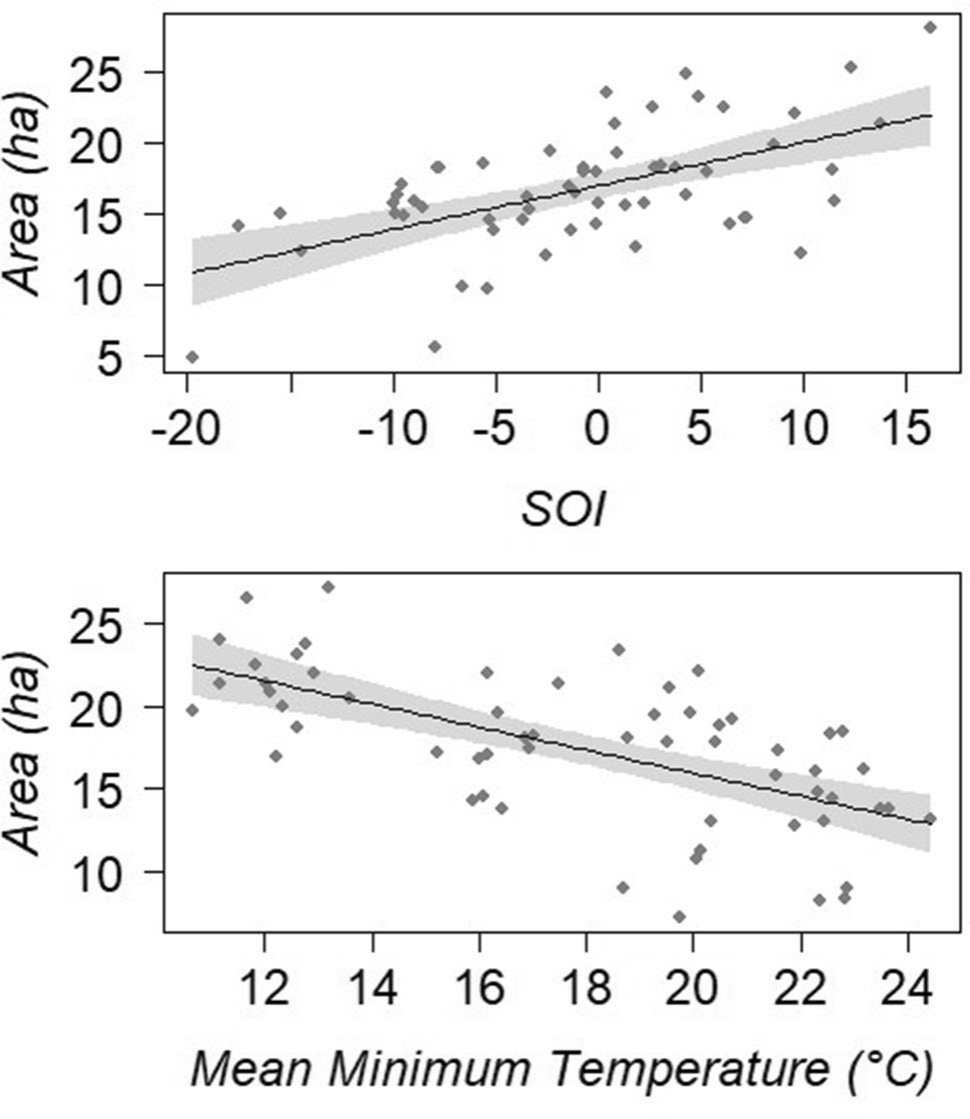
The GAMM analysis indicated the (top) SOI and (bottom) mean minimum temperature were the best predictors for area changes in mangrove canopy at Mangrove Bay (AICc = 326.69, r^2^(adj) = 0.508, t = 37.09, n = 58; SOI: F = 27.09, P = 0.0001; Mean Minimum Temperature: F = 36.95, P = 0.0001). Both variables displayed a linear relationship with the mangrove canopy area, with (top) SOI values indicative of El Nino and (bottom) mean minimum temperatures relating to lower green canopy.

The GAMMs analysis showed that the SOI intensity, and mean minimum temperature were strongly correlated to variation in green mangrove canopy area across the 29 years when analysed seasonally (AICc = 326.69, r^2^(adj) = 0.508, t = 37.09, n = 58; SOI: F = 27.09, P = 0.0001; Mean Minimum Temperature: F = 36.95, P = 0.0001) (Table 1 and SI6). SOI displayed a positive linear association with the canopy area, indicating that increases in green canopy cover occurred when conditions were not likely to be representative of El Niño events (Fig. 3). Whilst seasonal mean minimum temperature displayed a negative linear association with canopy, indicating the increases in green canopy occurred when seasonal mean minimum temperature was lower (Fig. 3).

**Table 1:** Variable importance from GAMMs.

Variable	Importance
SOI	0.999
Mean minimum temperature	0.401
Highest temperature	0.302
Mean maximum temperature	0.198
Lowest temperature	0.183
Highest minimum temperature	0.104
Lowest maximum temperature	0.097
Total precipitation	0.095

SOI and Mean Minimum temperature had highest importance, indicating they are the most strongly correlated to mangrove change at Mangrove Bay.

### Mangrove NDVI

**Figure 4:**
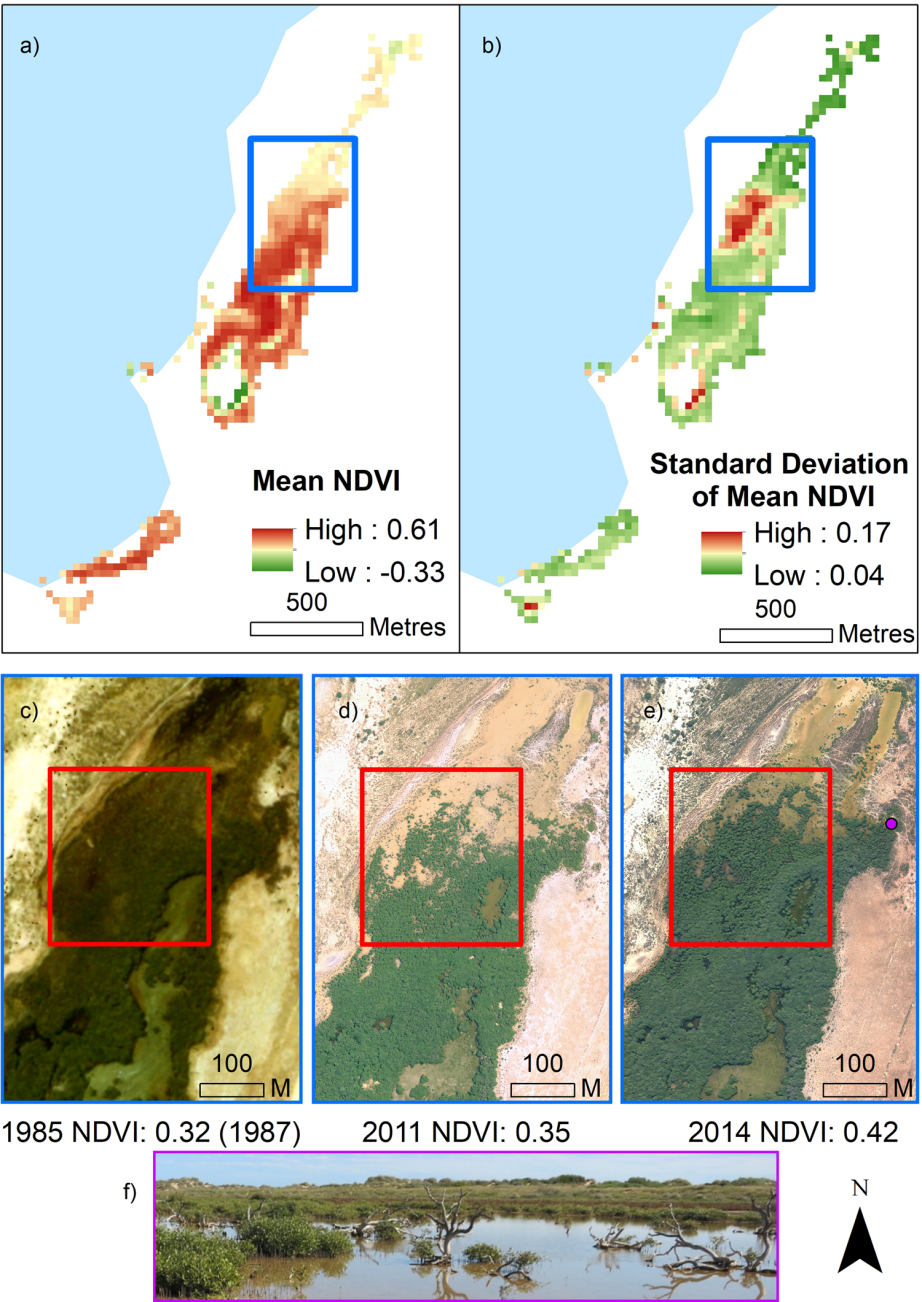
(**a**) Mean temporal NDVI across the Mangrove Bay site is highest in the centre of the mangrove stand, proximal to the lagoon entrance, where (**b**) standard deviation of mean NDVI is lowest. Spatial clustering of high standard deviation of mean NDVI is apparent in the northern section, at the edge of the mangrove canopy. This area (blue outline represents area of insets **c**–**e**) in the aerial images shows examples (red box) of canopy thinning and regrowth across the study period observed at the site. NDVI values correspond to the average values of the entire mangrove region (the whole site) for that year. Dieback of trees can be seen from the photo (**f**) which was captured on site in June 2014 adjacent to this section of the stand (indicated by purple circle in (**e**). Aerial imagery provided by WA Department of Biodiversity, Conservation and Attractions. For larger version (**c**) see SI10.

The mean temporal NDVI and its standard deviation was calculated for all pixels that were classified as mangrove from the above mangrove spectral classification. Spatial trends were apparent across the site (Fig. 4), with the edges of the core mangrove canopy area, particularly at the northern end of the region, exhibiting high variation in standard deviation of mean NDVI (Fig. 4), and lower mean NDVI. This area coincides with dead mangrove trees and subsequent new mangrove growth apparent in Fig. 4 and representative of the changes in green canopy area depicted in the spectral supervised classification (Fig. 2).

## Discussion

Climate change has heightened the need for increasing our understanding of how ocean-climate interactions influence the environmental variables and associated ecosystems. In addressing this issue our study focussed on the SOI, and variation in air temperature and precipitation. The SOI is known to influence factors that impose environmental constraints on mangroves in the NW region of Australia including sea level, and temperature, indicators we found to strongly correlate with mangrove canopy area across the study period. We were able to demonstrate the effect of SOI and high temperature on mangrove canopy at Mangrove Bay through analysis of twenty-nine years of near continuous Landsat data and provide a mechanism to further explore mangrove change relationships to climatic patterns. We found that during this study period mangrove canopy area at this NW Australian site exhibited a decreasing trend during periods of negative SOI, which are indicative of El Niño conditions.

The periods of negative SOI (El Niño conditions) coincide with low sea level, and low precipitation along the west Australian coast^14^. Previous studies have reported that high salinity, and low freshwater inputs from rainfall^25^, groundwater or river flows can lead to mangrove dieback, particularly in areas of restricted tidal flows^26^, or droughts^7,10^. With decreased inundation in arid conditions mangrove trees have been documented to progressively lose leaves with eventual death due to a diminished capacity for water uptake and salt exclusion^10^. El Niño conditions on the northern and west Australian coast appear to amplify this, with high levels of salinity associated with low sea level at this World Heritage Area site during two El Niño events^19^, and large-scale dieback also occurring at the Gulf of Carpentaria in northern Australia during the 2015–2016 El Niño event with low sea level, low rainfall, and high temperatures also recorded^7,16,27^. Both the Mangrove Bay and Gulf of Carpentaria sites occur within the northern Australia tropical region and have climate and other habitats influenced by ENSO (e.g., sea-surface temperature, coral bleaching). Whilst previous research is limited^5,16,19^ these dieback events suggest these areas have similar susceptibility to ENSO changes and in turn similar ecological responses were observed. Further research of stressors is required to ascertain if this response is apparent in other mangrove patches in this region.

Spatial differences in mangrove condition were illustrated by areas of high variance in canopy condition determined from NDVI. These areas were dynamic and illustrated tree death, and subsequent new growth as an oscillating pattern throughout the long-term study period (Fig. 2) and reflected earlier findings by Lovelock et al.^19^ at this site during two El Niño events that occurred during the time span of this study. Mangrove forest fragmentation has been associated with mangrove deforestation and loss of function, including changes in species composition^28^, the temporal NDVI spatial clustering metrics indicate that there are changes to the canopy that should be monitored further. The relationship with ENSO and the oscillating pattern in canopy area and condition displayed through our dataset shows that mangroves are sensitive to climate extremes though are able to recover during benign climatic periods, and in SOI phases indicative of La Niña conditions when sea level is higher in this region. The most spatially dynamic area of mangrove decline was found at the landward edge of the mangrove region, away from the creek entrance and lagoon. This area of the mangrove canopy has been shown to contain shorter trees compared to those close to the seaward side (Hickey et al. 2018), suggesting that this northern edge may be at the upper thresholds for optimal growth conditions in the area and therefore may be most sensitive to changes in environmental variables, such as a decline in sea level (associated with El Nino)^14^ and increase in air temperature as we report here.

Changes in environmental conditions due to SOI are common to many regions of the world, with conditions in marine and terrestrial systems affected, including rainfall and drought conditions (Harris et al. 2018), sea surface temperature and coral bleaching^29^. However, this relationship in mangrove environments has only recently begun to be explored. Limited studies tend to focus on small locations within the mangrove canopy and not across the entire mangrove area, or on a singular event and not across a significant time-period covering both impact and recovery of mangrove canopy alongside multiple ENSO phases, as we do here. This study shows that the benign climatic phases are essential for recovery to occur. However, anthropogenic climate change has been shown to affect environmental variables such as increased temperature and change in rainfall patterns^30^. Such variations in temperature, or rainfall may alter the conditions during the benign climatic phase, potentially limiting the recovery opportunity for mangroves. This may further increase the vulnerability of these arid and semi-arid mangroves already near physiological thresholds, during sustained negative SOI impact events (that is El Niño conditions), such as increasing the area of mortality and decline in canopy condition^16^, or species composition^28^. The importance of SOI associated reductions in sea level and increased temperature warrants a larger investigation of the fate of mangroves in Australia, especially with predicted changes in aridity^31^ within tropical and subtropical regions, and the intensification of the effects of ENSO in the tropical Pacific and eastern Indian Ocean regions^12,13,32^.

Ecosystem structure, function, and species distribution are greatly influenced by climate extremes^33^. Further analysis of ecosystem-scale threshold responses elsewhere would build on the findings of this study and may provide information on the duration and level of variation in sea level and temperature increase required before canopy loss or dieback occurs, whilst also providing insight into recovery time, and areas of the mangrove stand that are most vulnerable. Undertaking such studies enables for enhanced anticipation of the response of mangroves to changes in climate, including the frequency and intensity of climate extremes^34^.

This study has shown that the SOI is strongly correlated to mangrove changes at Mangrove Bay, a site located within the World Heritage Area of Ningaloo Marine Park. This site, while relatively small, is representative of mangrove sites in arid and semi-arid regions. The NW Australian coastline, like other arid locations has mangrove trees that are small in stature and forests with limited extent, though they function as important habitats for fish and prawns, as well as other marine fauna in these regions. The long temporal record at Mangrove Bay has illustrated that ENSO conditions affect mangrove canopy area and ‘greenness’, which has been detected using free Landsat imagery, alongside climate variables. This method could be applied to other arid and semi-arid mangroves, or those mangrove stands located within tropical regions affected by ENSO conditions to further establish the relationship between mangroves condition and ENSO. This information may be used in management, to establish monitoring sites, and to minimise hydrologic constraints from anthropogenic activities during periods when sea level is lower due to ENSO and when mangroves may be experiencing reduced tidal connectivity and freshwater inundation.

## Methods

### Mangrove classification

Cloud-free Landsat 5, 7 TM and 8 OLI imagery (path 115, row 75) were acquired for all available months from 1987 to 2016 (*n* = 213) where there was no cloud, striping, atmospheric interference (SI8). Corrections were performed to convert the pixels (30 × 30 m) to top of atmosphere reflectance with the bands utilised (corrected for sun angle)^31–37^. A combination of unsupervised and supervised classification within ERDAS Imagine was used to categorise the mangrove canopy within the site (SI2). This was done separately for Landsat 8 OLI to account for the difference in band wavelengths (SI3). The site was classified into 7 classes and accuracy assessment was undertaken for 11 reference dates where high resolution orthorectified aerial imagery was available (SI5). Orthorectified aerial imagery was subdivided into 30 m grid cells that corresponded with Landsat scene pixels. Each cell was assigned to a class (SI5). Identification of cell as mangrove canopy, required dense mangrove surface area, or at least 80% surface coverage (SI5). This study presents the mangrove canopy classification which yielded the strongest accuracy assessment of the classes (SI5). Assessment in the form of kappa coefficients was used to assess the level of agreement for all reference dates (SI5)^38^. For each scene, pixels identified as mangrove comprised the mangrove canopy area.

### Mangroves, climatic variables and the SOI

Temperature and SOI variables were accessed from the Bureau of Meteorology (BOM) (http://www.bom.gov.au/climate/averages/tables/cw_005007.shtml). Temperature observations were recorded at Learmonth station (Latitude: − 22.24 Longitude: 114.10; Height of station above mean sea level (metres: 5) and seasonal statistics were generated from monthly values (mean minimum, mean maximum, lowest maximum, highest minimum). Seasonal mean SOI was generated from BOM data (http://www.bom.gov.au/climate/enso/soi/). A GAMM analysis was utilised within the R statistical package v.3.4.1, accessing the mgcv and MuMIn libraries to ascertain the relationship between air temperature derivatives (SI1), precipitation and the SOI, with mangrove canopy area. A GAMM analysis was utilised as it can fit both linear and non-linear responses in mixed effect models. Variables were checked for collinearity and were not highly correlated (correlation coefficients < 0.7). Overdispersion of data was checked a priori and normality of residuals in posthoc and neither showed significant deviation. All possible combinations of predictors were considered. The Gaussian model was applied to the data analysis. GAMM provided a flexible robust method to analyse the independent effects of environmental variables whilst also accounting for the temporal aspect. They allow for complex responses due to utilising non-parametric smoothing functions^39^. Time was treated as a random variable to account for potential temporal autocorrelation. The time variables used was year & season. The model with the fewest variables and within 2 AICc (Akaike Information Criterion corrected for finite samples) of the lowest AICc for all possible models was determined to be the best model^40^. A backward stepwise model was utilised for GAMM analysis. Relative importance of each environmental variable was calculated for each variable by summing the model weighted AICc values for all model combinations (maximum 4 variables) for each model where the variable was included^41^ (Table 1; SI6). Analysis was conducted using the R language packages mgcv and MuMIn using the function ‘importance’.

### Mangrove NDVI

The mangrove boundary was determined as any pixel that was classified as mangrove from the spectral classification. Clear misclassified pixels (e.g., in water) were removed (5 pixels). The NVDI was calculated for each Landsat scene (SI7) for this mangrove boundary. NDVI is strongly correlated to canopy closure in mangroves (*r*^*2*^ = 0.91)^24^ and is often used as a proxy for measuring mangrove canopy condition, or ‘greenness’^5,19,35–45^, including in arid mangrove stands^5,19,46^. This is as it measures the absorbance of chlorophyll in the red band and the reflection of the mesophyll in the near-infrared band^47^. Values range from − 1 to + 1, where green vegetation corresponds to values above zero, and dense closed canopies are closer to 1^44^. The temporal mean NDVI for each pixel was used to provide a spatial indication of mangrove canopy condition and identify clusters of high and low variances (standard deviation) in temporal mean NDVI to investigate any spatial patterns of variance.

## Supplementary Information

Supplementary Information.
