# Supplementary material for: ENSO feedback drives variations in dieback at a marginal mangrove site
Source: Sci Rep. 2021 Apr 14;11:8130. doi: 10.1038/s41598-021-87341-5 (PMC8046780; doi:10.1038/s41598-021-87341-5)
Supplement: Supplementary file 1 — Supplementary Information. [file 41598_2021_87341_MOESM1_ESM.pdf]

Supplementary Information

ENSO feedback drives variations in dieback for a marginal mangrove site

Hickey, S. M.[1,2,3*], Radford B.[1,2,3], Callow, J. N.[1], Phinn, S. R.[4], Duarte, C.M.[5], Lovelock, C.E.[6]

[1]The School of Agriculture and Environment, Faculty of Science, University of Western Australia, Perth, WA, Australia

[2]The Oceans Institute, University of Western Australia, Perth, WA, Australia

[3]Australian Institute of Marine Science (AIMS), Crawley, WA, Australia

[4]Remote Sensing Research Centre, School of Earth and Environmental Sciences, The University of Queensland, Brisbane, Queensland, Australia

[5]Red Sea Research Center, King Abdullah University of Science and Technology, Thuwal, Saudi Arabia

[6]School of Biological Sciences, The University of Queensland, Brisbane, Queensland, Australia

**corresponding author: sharyn.hickey@uwa.edu.au*

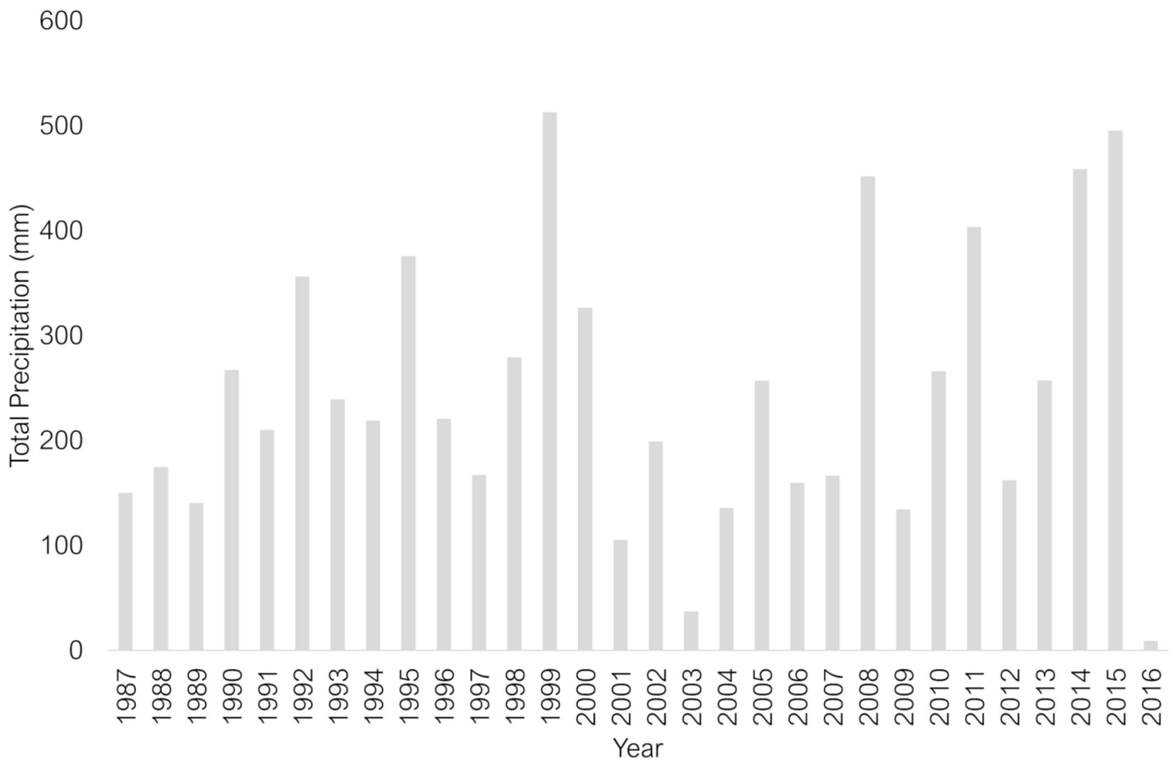

SI1 Figure A: Total annual precipitation recorded for Mangrove Bay (measured at Learmonth Bureau of Meteorology station) used in GAMM analysis. Low precipitation in 2003 coincided with El Nino and mangrove dieback at the site (Lovelock et al., 2017).

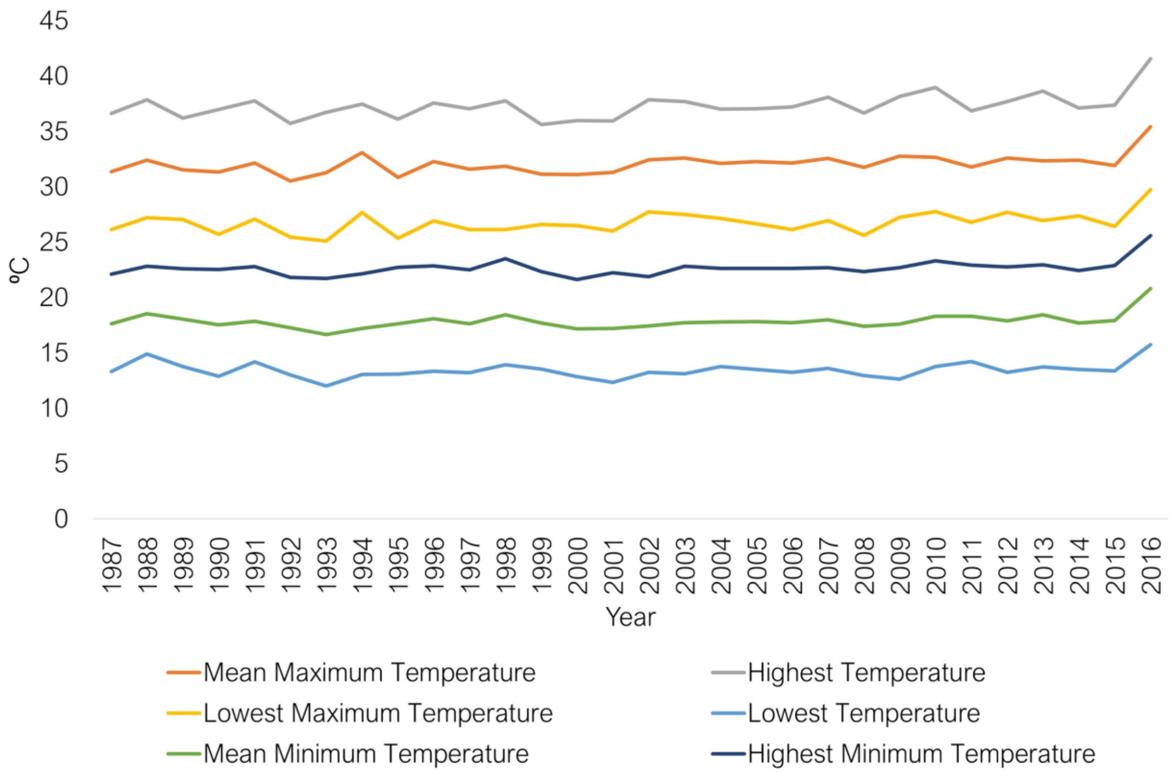

SI1 Figure B: Annual averages of temperature variables used in GAMM analysis recorded for Mangrove Bay (measured at Learmonth Bureau of Meteorology station).

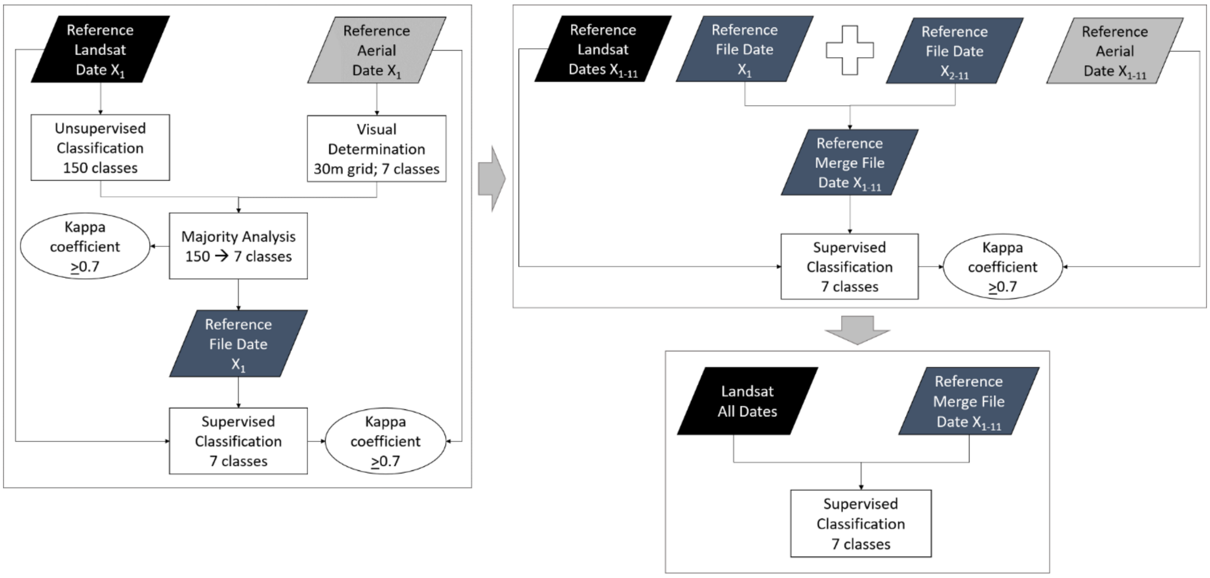

Figure SI2: Conceptual diagram showing the process of mangrove classification, showing initial unsupervised classification and supervised classification. A maximum likelihood algorithm was used. Accuracy assessment was undertaken using kappa coefficients (see Table).

Table SI3: Bands and wavelengths utilised in the mangrove classification for Landsat 5, 7 TM and 8 OLI.

**Bands Landsat 5 and 7**	**Landsat 5 TM Wavelength (micrometers)**	**Landsat 7 TM Wavelength (micrometers)**	**Bands Landsat 8**	**Landsat 8 OLI Wavelength (micrometers)**
Band 1 - Blue	0.45-0.52	0.45-0.52	Band 2 - Blue	0.452 - 0.512
Band 2 - Green	0.52-0.60	0.52-0.60	Band 3 - Green	0.533 - 0.590
Band 3 - Red	0.63-0.69	0.63-0.69	Band 4 - Red	0.636 - 0.673
Band 4 - Near Infrared (NIR)	0.76-0.90	0.77-0.90	Band 5 - Near Infrared (NIR)	0.851 - 0.879
Band 5 - Shortwave Infrared (SWIR) 1	1.55-1.75	1.55-1.75	Band 6 - Shortwave Infrared (SWIR) 1	1.566 - 1.651
Band 7 - Shortwave Infrared (SWIR) 2	2.08-2.35	2.09-2.35	Band 7 - Shortwave Infrared (SWIR) 2	2.107 - 2.294

SI4: Description of classes used to classify the Mangrove bay site from aerial and Landsat scenes. Only the class, mangrove is analysed in this study.

Vegetation Type	Description
Mangrove	Dense mangrove trees, or trees covering 80% surface area
Mangrove/Saltmarsh	Combined area of mangrove and saltmarsh. Mangrove is the majority component (>50%)
Other vegetation/sand	Combined area of coastal grasses on sand
Mangrove/ saltmarsh/saltflat/sand (may include coastal grasses)	Combined area of saltflat, saltmarsh and mangrove
Saltflat/sand	Saltflat or sand area that is adjacent to mangroves and/or saltmarsh but has minimal (<20%) vegetation
Water	Water including adjacent coast, and internal lagoons
Mangrove/Water	Combined mangrove and lagoon area. May also be mangroves fringing coast, or lagoon entrance

SI5: Results of the accuracy assessment of mangrove classification. Error matrices of supervised classification with reference data. Error matrices were carried out on all pixels (30mx30m) (n_{pixels} = *8400)* within study region. Kappa coefficients and overall accuracy show strong agreement across reference data and supervised classification.

Landsat Scene Date (Landsat series)	Orthorectified Aerial Image Date	Error Measurement (%)		Kappa	overall accuracy
		Agreement	93.27		
May-1987 (5)	Mar-1985	Omission	6.73	0.82	88.37
		Commission	32.64		
		Agreement	90.48		
Dec-1999 (5)	Aug-2000	Omission	9.52	0.78	85.30
		Commission	29.15		
		Agreement	94.34		
Jul-2003 (5)	Jul-2003	Omission	5.66	0.80	86.92
		Commission	34.64		
		Agreement	95.00		
Oct-2006 (5)	Oct-2006	Omission	5.00	0.79	86.32
		Commission	39.77		
		Agreement	91.22		
Aug-2007 (5)	Aug-2007	Omission	8.78	0.71	80.06
		Commission	20.86		
		Agreement	96.54		
Nov-2008 (5)	Nov-2008	Omission	3.46	0.76	83.55
		Commission	26.40		
		Agreement	95.40		
Dec-2010 (5)	Oct-2010	Omission	4.60	0.81	87.19
		Commission	22.19		
		Agreement	83.96		
Jun-2011 (5)	Jul-2011	Omission	16.04	0.80	86.49
		Commission	15.51		
		Agreement	81.86		
Aug-2000 (7)	Aug-2000	Omission	18.14	0.85	90.49
		Commission	21.08		
		Agreement	91.18		
Sept-2013 (8)	Sept-2013	Omission	8.82	0.80	85.63
		Commission	13.55		
		Agreement	93.12		
Jul-2014 (8)	Jul-2014	Omission	6.89	0.80	86.35
		Commission	19.12		

SI6: AICc results of GAMMs analysis (top 10 models). + indicates the variable is included in the model. The best model is 1: SOI and mean minimum temperature indicated by the lowest AICc score, and lowest number of variables. The model with only the intercept is included.

Model Number	Intercept	Highest Minimum Temperature	Highest Temperature	Lowest Maximum Temperature	Lowest Temperature	Mean Maximum Temperature	Mean Minimum Temperature	Total Precipitation	SOI	AIC	df	logLik	AICc	delta	weight
1	17.27508621	NA	NA	NA	NA	NA	+	NA	+	324.4473	7	-155	326.6873	0.0000	0.2444
2	17.27508621	NA	+	NA	NA	NA	NA	NA	+	325.2180	7	-156	327.4580	0.7707	0.1663
3	17.27508621	NA	NA	NA	NA	+	NA	NA	+	326.1343	7	-156	328.3743	1.6870	0.1051
4	17.27508621	NA	NA	NA	+	NA	NA	NA	+	326.2381	7	-156	328.4781	1.7908	0.0998
5	17.27508621	+	NA	NA	NA	NA	+	NA	+	327.0471	9	-155	330.7971	4.1098	0.0313
6	17.27508621	NA	+	NA	NA	NA	NA	+	+	327.0772	9	-155	330.8272	4.1398	0.0308
7	17.27508621	NA	+	NA	NA	NA	+	NA	+	327.1768	9	-155	330.9268	4.2394	0.0293
8	17.27508621	NA	NA	+	NA	NA	NA	NA	+	328.8892	7	-157	331.1292	4.4418	0.0265
9	17.27508621	NA	NA	NA	NA	+	+	NA	+	327.8737	9	-155	331.6237	4.9363	0.0207
10	17.27508621	+	NA	NA	NA	NA	NA	NA	+	329.5205	7	-158	331.7605	5.0731	0.0193
Intercept	17.27508621	NA	NA	NA	NA	NA	NA	NA	NA	359.6131	3	-177	360.0575	33.3702	0.0000

SI7: Equation 1: Normalised Difference Vegetation Index (NDVI). Refer to Table SI3 for band and wavelength information.

NDVI = NIR band – Red band / NIR band + Red band *(Equation 1)*

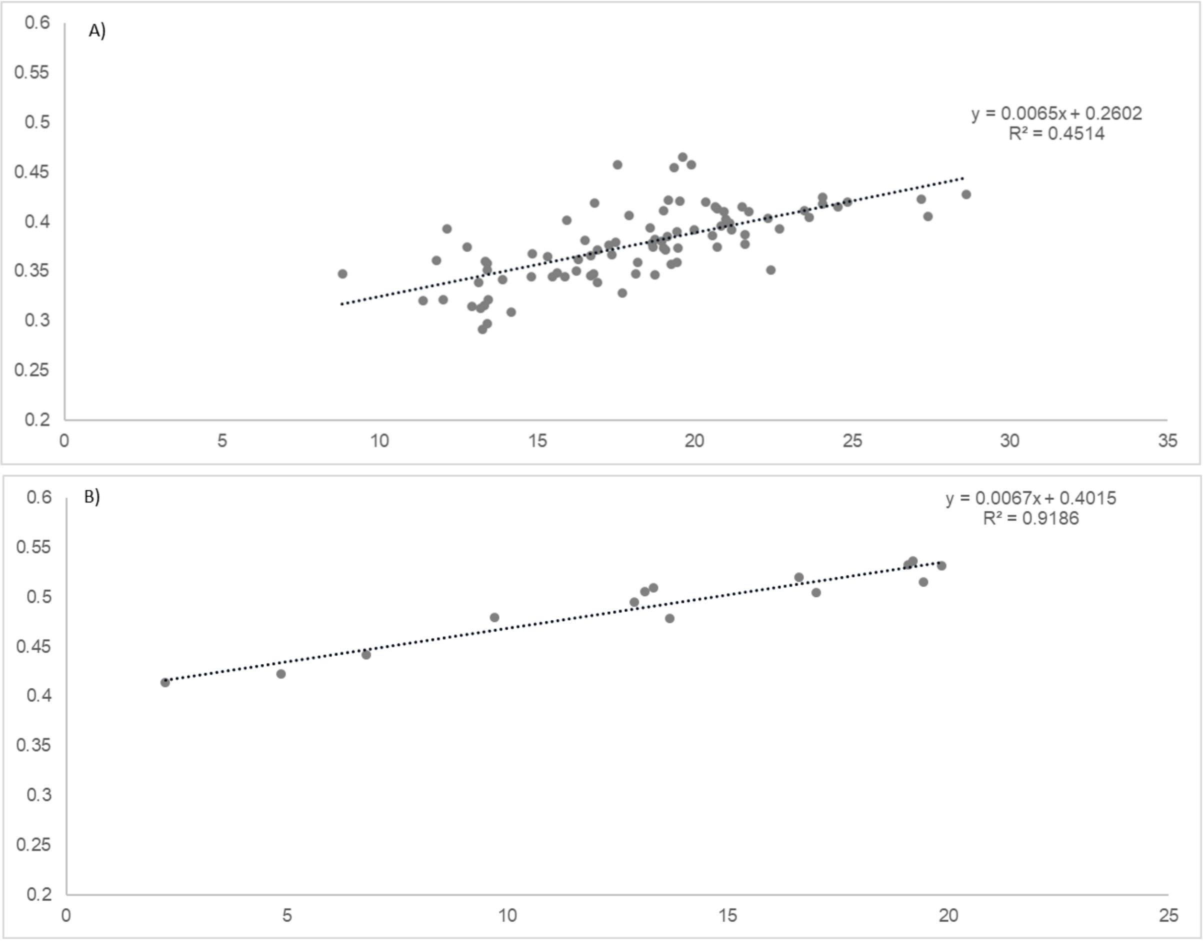

Figure SI8: Relationship between mangrove NDVI and mangrove area for each time-point. NDVI and area are closely related, with NDVI increasing linearly when area increases. Landsat 5, 7 derived data shown in A, and Landsat 8 derived data in B.

SI9: Landsat Scenes (n=213) used in analysis. * denotes reference layer used in accuracy assessment (n=11) (Table 2).

Landsat	Month	Year	Landsat Scenes
Landsat 5	5	1987	LT51150751987149ASA00*
Landsat 5	8	1987	LT51150751987229ASA00
Landsat 5	9	1987	LT51150751987261ASA00
Landsat 5	10	1987	LT51150751987293ASA00
Landsat 5	11	1987	LT51150751987325ASA00
Landsat 5	12	1987	LT51150751987341ASA00
Landsat 5	1	1988	LT51150751988008ASA00
Landsat 5	2	1988	LT51150751988056ASA00
Landsat 5	3	1988	LT51150751988072ASA00
Landsat 5	4	1988	LT51150751988104ASA00
Landsat 5	5	1988	LT51150751988152ASA00
Landsat 5	6	1988	LT51150751988168ASA00
Landsat 5	7	1988	LT51150751988200ASA00
Landsat 5	8	1988	LT51150751988232ASA00
Landsat 5	9	1988	LT51150751988264ASA00
Landsat 5	10	1988	LT51150751988296ASA00
Landsat 5	11	1988	LT51150751988328ASA00
Landsat 5	12	1988	LT51150751988360ASA00
Landsat 5	1	1989	LT51150751989026ASA00
Landsat 5	5	1989	LT51150751989138ASA00
Landsat 5	6	1989	LT51150751989170ASA00
Landsat 5	7	1989	LT51150751989186ASA00
Landsat 5	8	1989	LT51150751989218ASA01
Landsat 5	9	1989	LT51150751989266ASA00
Landsat 5	10	1989	LT51150751989282ASA00
Landsat 5	11	1989	LT51150751989330ASA00
Landsat 5	12	1989	LT51150751989362ASA00
Landsat 5	4	1990	LT51150751990109ASA00
Landsat 5	5	1990	LT51150751990141ASA00
Landsat 5	6	1990	LT51150751990173ASA00
Landsat 5	7	1990	LT51150751990205ASA00
Landsat 5	8	1990	LT51150751990237ASA00
Landsat 5	9	1990	LT51150751990253ASA00
Landsat 5	12	1990	LT51150751990349ASA00
Landsat 5	2	1991	LT51150751991048ASA00
Landsat 5	5	1991	LT51150751991128ASA00
Landsat 5	7	1991	LT51150751991192ASA00
Landsat 5	8	1991	LT51150751991224ASA00

Landsat 5	9	1991	LT51150751991256ASA00
Landsat 5	10	1991	LT51150751991288ASA00
Landsat 5	11	1991	LT51150751991320ASA00
Landsat 5	12	1991	LT51150751991336ASA00
Landsat 5	1	1992	LT51150751992003ASA00
Landsat 5	2	1992	LT51150751992035ASA00
Landsat 5	3	1992	LT51150751992067ASA00
Landsat 5	4	1992	LT51150751992099ASA00
Landsat 5	6	1992	LT51150751992179ASA00
Landsat 5	7	1992	LT51150751992195ASA00
Landsat 5	8	1992	LT51150751992227ASA00
Landsat 5	9	1992	LT51150751992259ASA00
Landsat 5	12	1992	LT51150751992339ASA00
Landsat 5	3	1993	LT51150751993069ASA00
Landsat 5	4	1993	LT51150751993117ASA00
Landsat 5	6	1993	LT51150751993165ASA00
Landsat 5	7	1993	LT51150751993197ASA00
Landsat 5	8	1993	LT51150751993229ASA00
Landsat 5	9	1993	LT51150751993245ASA00
Landsat 5	10	1993	LT51150751993277ASA00
Landsat 5	11	1993	LT51150751993325ASA00
Landsat 5	1	1994	LT51150751994008ASA00
Landsat 5	2	1994	LT51150751994040ASA00
Landsat 5	3	1994	LT51150751994072ASA01
Landsat 5	5	1994	LT51150751994136ASA00
Landsat 5	6	1994	LT51150751994168ASA00
Landsat 5	7	1994	LT51150751994184ASA00
Landsat 5	8	1994	LT51150751994232ASA00
Landsat 5	9	1994	LT51150751994248ASA00
Landsat 5	10	1994	LT51150751994296ASA00
Landsat 5	11	1994	LT51150751994312ASA00
Landsat 5	1	1995	LT51150751995011ASA00
Landsat 5	3	1995	LT51150751995075ASA00
Landsat 5	4	1995	LT51150751995107ASA00
Landsat 5	5	1995	LT51150751995139ASA00
Landsat 5	6	1995	LT51150751995155ASA00
Landsat 5	7	1995	LT51150751995203ASA00
Landsat 5	8	1995	LT51150751995219ASA00
Landsat 5	10	1995	LT51150751995299ASA00
Landsat 5	11	1995	LT51150751995315ASA00
Landsat 5	12	1995	LT51150751995363ASA00
Landsat 5	2	1996	LT51150751996046ASA00

Landsat 5	3	1996	LT51150751996078ASA00
Landsat 5	4	1996	LT51150751996094ASA00
Landsat 5	5	1996	LT51150751996142ASA00
Landsat 5	6	1996	LT51150751996174ASA00
Landsat 5	8	1996	LT51150751996222ASA00
Landsat 5	9	1996	LT51150751996270ASA00
Landsat 5	10	1996	LT51150751996286ASA00
Landsat 5	11	1996	LT51150751996318ASA00
Landsat 5	1	1997	LT51150751997016ASA00
Landsat 5	3	1997	LT51150751997064ASA00
Landsat 5	4	1997	LT51150751997112ASA00
Landsat 5	5	1997	LT51150751997144ASA00
Landsat 5	7	1997	LT51150751997192ASA00
Landsat 5	8	1997	LT51150751997224ASA00
Landsat 5	10	1997	LT51150751997304ASA00
Landsat 5	12	1997	LT51150751997352ASA00
Landsat 5	1	1998	LT51150751998003ASA00
Landsat 5	2	1998	LT51150751998035ASA00
Landsat 5	3	1998	LT51150751998067ASA00
Landsat 5	4	1998	LT51150751998115ASA00
Landsat 5	6	1998	LT51150751998179ASA00
Landsat 5	8	1998	LT51150751998243ASA00
Landsat 5	9	1998	LT51150751998259ASA00
Landsat 5	10	1998	LT51150751998291ASA00
Landsat 5	11	1998	LT51150751998323ASA00
Landsat 5	12	1998	LT51150751998355ASA00
Landsat 5	2	1999	LT51150751999054ASA00
Landsat 5	3	1999	LT51150751999070ASA00
Landsat 5	4	1999	LT51150751999102ASA00
Landsat 5	6	1999	LT51150751999166ASA00
Landsat 5	7	1999	LT51150751999198ASA00
Landsat 5	8	1999	LT51150751999230ASA00
Landsat 5	9	1999	LT51150751999262ASA00
Landsat 5	10	1999	LT51150751999294ASA00
Landsat 5	11	1999	LT51150751999310ASA00
Landsat 5	12	1999	LT51150751999358ASA00*
Landsat 7	1	2000	LE71150752000017SGS00
Landsat 7	3	2000	LE71150752000065ASA00
Landsat 7	4	2000	LE71150752000097ASA00
Landsat 7	8	2000	LE71150752000225ASA00*
Landsat 7	12	2000	LE71150752000337ASA00
Landsat 7	5	2001	LE71150752001131ASA00

Landsat 7	8	2001	LE71150752001243ASA00
Landsat 7	9	2001	LE71150752001259ASA00
Landsat 7	2	2002	LE71150752002038ASA00
Landsat 7	3	2002	LE71150752002070ASA00
Landsat 7	4	2002	LE71150752002102SGS00
Landsat 7	7	2002	LE71150752002198ASA00
Landsat 7	12	2002	LE71150752002342ASA00
Landsat 7	3	2003	LE71150752003089ASA00
Landsat 5	7	2003	LT51150752003193ASA01*
Landsat 5	8	2003	LT51150752003225ASA01
Landsat 5	9	2003	LT51150752003257ASA01
Landsat 5	10	2003	LT51150752003289ASA01
Landsat 5	11	2003	LT51150752003305ASA01
Landsat 5	12	2003	LT51150752003353ASA01*
Landsat 5	1	2004	LT51150752004020ASA01
Landsat 5	2	2004	LT51150752004036ASA01
Landsat 5	3	2004	LT51150752004068ASA01
Landsat 5	4	2004	LT51150752004116ASA01
Landsat 5	5	2004	LT51150752004132ASA01
Landsat 5	6	2004	LT51150752004164XXX01
Landsat 5	8	2004	LT51150752004228ASA00
Landsat 5	9	2004	LT51150752004260ASA00
Landsat 5	10	2004	LT51150752004292ASA00
Landsat 5	11	2004	LT51150752004324ASA00
Landsat 5	12	2004	LT51150752004356ASA00
Landsat 5	1	2005	LT51150752005022ASA02
Landsat 5	2	2005	LT51150752005054ASA01
Landsat 5	6	2005	LT51150752005166ASA01
Landsat 5	7	2005	LT51150752005198ASA01
Landsat 5	8	2005	LT51150752005230ASA01
Landsat 5	9	2005	LT51150752005262ASA01
Landsat 5	10	2005	LT51150752005278ASA01
Landsat 5	11	2005	LT51150752005310ASA01
Landsat 5	2	2006	LT51150752006041ASA00
Landsat 5	4	2006	LT51150752006105ASA00
Landsat 5	5	2006	LT51150752006137ASA01
Landsat 5	6	2006	LT51150752006169ASA01
Landsat 5	7	2006	LT51150752006201ASA00
Landsat 5	8	2006	LT51150752006217ASA00
Landsat 5	9	2006	LT51150752006265ASA00
Landsat 5	10	2006	LT51150752006281ASA00*
Landsat 5	11	2006	LT51150752006313ASA00

Landsat 5	12	2006	LT51150752006345ASA00
Landsat 5	1	2007	LT51150752007028ASA00
Landsat 5	3	2007	LT51150752007060ASA00
Landsat 5	4	2007	LT51150752007108ASA00
Landsat 5	6	2007	LT51150752007172ASA00
Landsat 5	7	2007	LT51150752007188ASA00
Landsat 5	8	2007	LT51150752007220ASA00*
Landsat 5	9	2007	LT51150752007252ASA00
Landsat 5	2	2008	LT51150752008047ASA00
Landsat 5	10	2008	LT51150752008287ASA00
Landsat 5	11	2008	LT51150752008319ASA00*
Landsat 5	3	2009	LT51150752009081ASA00
Landsat 5	5	2009	LT51150752009129ASA00
Landsat 5	10	2009	LT51150752009289ASA00
Landsat 5	1	2010	LT51150752010020ASA00
Landsat 5	2	2010	LT51150752010052ASA00
Landsat 5	5	2010	LT51150752010132ASA00
Landsat 5	6	2010	LT51150752010180ASA00
Landsat 5	12	2010	LT51150752010356ASA00*
Landsat 5	4	2011	LT51150752011119ASA00
Landsat 5	6	2011	LT51150752011167ASA00*
Landsat 8	4	2013	LC81150752013108LGN01
Landsat 8	6	2013	LC81150752013172LGN00
Landsat 8	7	2013	LC81150752013188LGN00
Landsat 8	8	2013	LC81150752013220LGN00
Landsat 8	9	2013	LC81150752013252LGN00*
Landsat 8	11	2013	LC81150752013316LGN00
Landsat 8	12	2013	LC81150752013348LGN00
Landsat 8	2	2014	LC81150752014047LGN00
Landsat 8	3	2014	LO81150752014063LGN00
Landsat 8	4	2014	LC81150752014095LGN00
Landsat 8	5	2014	LC81150752014143LGN00
Landsat 8	7	2014	LC81150752014191LGN00*
Landsat 8	8	2014	LC81150752014223LGN00
Landsat 8	9	2014	LC81150752014255LGN00
Landsat 8	10	2014	LC81150752014287LGN00
Landsat 8	11	2014	LC81150752014319LGN00
Landsat 8	12	2014	LC81150752014351LGN00
Landsat 8	2	2015	LC81150752015034LGN00
Landsat 8	4	2015	LC81150752015114LGN00
Landsat 8	5	2015	LC81150752015130LGN00
Landsat 8	6	2015	LC81150752015162LGN00

Landsat 8	7	2015	LC81150752015210LGN00
Landsat 8	9	2015	LC81150752015258LGN00
Landsat 8	10	2015	LC81150752015274LGN00
Landsat 8	12	2015	LC81150752015354LGN00
Landsat 8	2	2016	LC81150752016037LGN01
Landsat 8	3	2016	LC81150752016069LGN00
Landsat 8	5	2016	LC81150752016133LGN00

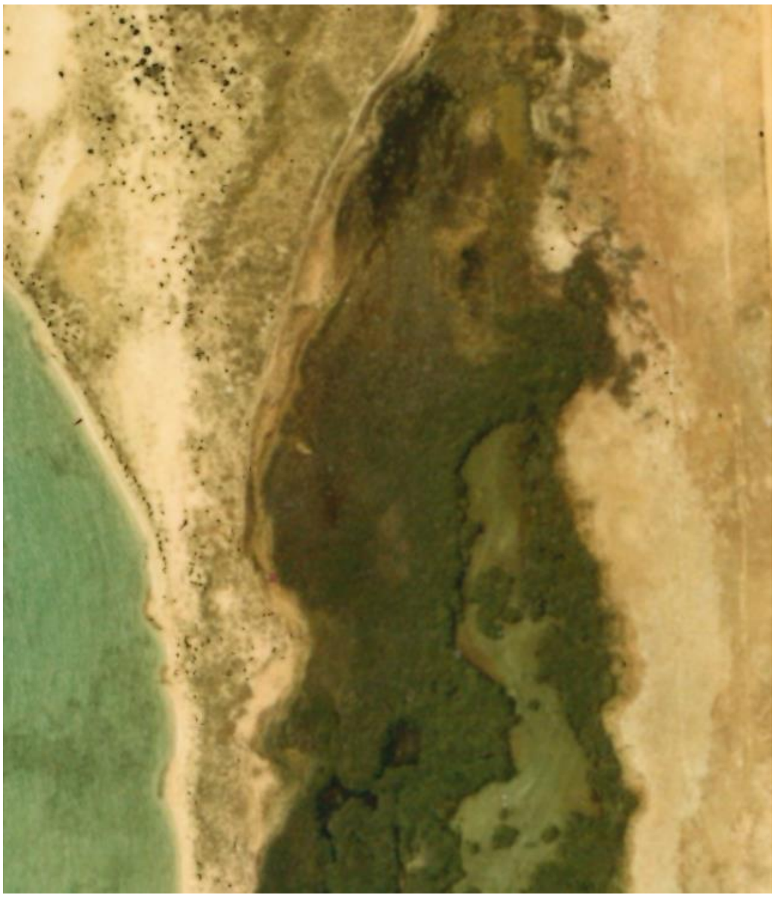

Figure SI 10: A close-up of the 1985 image prior to georectification to highlight the loss of green canopy apparent in the top left section of the forest. See Figure 4 for scale and location information.
